# Single-cell mapping of N6-methyladenosine in esophageal squamous cell carcinoma and exploration of the risk model for immune infiltration

**DOI:** 10.3389/fendo.2023.1155009

**Published:** 2023-03-21

**Authors:** Yuanliu Nie, Guangyue Yao, Xiaoying Xu, Yi Liu, Ke Yin, Jingjiang Lai, Qiang Li, Fengge Zhou, Zhe Yang

**Affiliations:** ^1^ Tumor Research and Therapy Center, Shandong Provincial Hospital, Shandong University, Jinan, Shandong, China; ^2^ Shandong First Medical University, College of Basic Medicine, Shandong First Medical University-Shandong Academy of Medical Sciences, Jinan, Shandong, China; ^3^ Department of Computer Science and Technology, Ocean University of China, Qingdao, China; ^4^ Department of Pathology, Shandong Provincial Hospital, Shandong University, Jinan, China; ^5^ Department of Oncology, Shandong Provincial Hospital Affiliated to Shandong First Medical University, Jinan, China; ^6^ Tumor Research and Therapy Center, Shandong Provincial Hospital Affiliated to Shandong First Medical University, Jinan, Shandong, China

**Keywords:** N6-Methyladenosine, esophageal squamous cell carcinoma, single-gene sequencing, immune infiltration, bioinfomatics

## Abstract

**Background:**

N6-methyladenosine (m6A) modification is the most common RNA modification, but its potential role in the development of esophageal cancer and its specific mechanisms still need to be further investigated.

**Methods:**

Bulk RNA-seq of 174 patients with esophageal squamous carcinoma from the TCGA-ESCC cohort, GSE53625, and single-cell sequencing data from patients with esophageal squamous carcinoma from GSE188900 were included in this study. Single-cell analysis of scRNA-seq data from GSE188900 of 4 esophageal squamous carcinoma samples and calculation of PROGENy scores. Demonstrate the scoring of tumor-associated pathways for different cell populations. Cell Chat was calculated for cell populations. thereafter, m6A-related differential genes were sought and risk models were constructed to analyze the relevant biological functions and impact pathways of potential m6A genes and their impact on immune infiltration and tumor treatment sensitivity in ESCC was investigated.

**Results:**

By umap downscaling analysis, ESCC single-cell data were labelled into clusters of seven immune cell classes. Cellchat analysis showed that the network interactions of four signaling pathways, MIF, AFF, FN1 and CD99, all showed different cell type interactions. The prognostic risk model constructed by screening for m6A-related differential genes was of significant value in the prognostic stratification of ESCC patients and had a significant impact on immune infiltration and chemotherapy sensitivity in ESCC patients.

**Conclusion:**

In our study, we explored a blueprint for the distribution of single cells in ESCC based on m6A methylation and constructed a risk model for immune infiltration analysis and tumor efficacy stratification in ESCC on this basis. This may provide important potential guidance for revealing the role of m6A in immune escape and treatment resistance in esophageal cancer.

## Introduction

Esophageal cancer is a common malignancy worldwide, ranking seventh in incidence and sixth in mortality of all malignancies (1). Esophageal cancer is mainly classified into esophageal squamous cell carcinoma (ESCC) and esophageal Adenocarcinoma (EAC), with ESCC being the most common histological type of esophageal cancer. In Asian countries, squamous cell carcinoma of the esophagus, accounts for approximately 95% of esophageal cancers (2). Despite significant breakthroughs in the diagnosis and treatment of esophageal squamous carcinoma (3), the prognosis of patients with esophageal squamous carcinoma remains poor, with a 5-year survival rate of less than 15% (4), largely because its pathogenesis has not been fully elucidated. With the further analysis of tumor development mechanism, a variety of therapies based on new tumor immune and metabolic targets (immunotherapy and targeted therapy, etc.) have emerged to improve the efficacy, but the prognosis of esophageal cancer patients is still poor (5), therefore, in-depth study of the mechanisms related to the development of esophageal cancer and the search for new effective therapeutic targets are important means to improve the overall survival rate of esophageal cancer patients. At the same time, an in-depth understanding of tumor heterogeneity from a genetic perspective is more conducive to dissecting the intrinsic features of ESCC.

N6-methyladenosine, (m6A) modification is one of the most common RNA modifications (6). m6A methyltransferase-like 3 (METTL3) is the most important component of m6A methyltransferase (7). According to recent studies, m6A methylation has been associated with a variety of human cancers, including cervical, colorectal, ovarian and lung cancers (8–11). m6A methylation is closely involved in cancer cell proliferation, apoptosis, invasion and migration, autophagy and metabolism. as well as metabolism and other biological processes (12–16). However, its biological role and molecular mechanisms in the development of esophageal squamous carcinoma are relatively limited. The m6A methylation modification has an important regulatory role in the proliferation, migration and invasion of esophageal cancer cells, and is of great research value and clinical significance in predicting the prognosis and targeting therapy of esophageal cancer. However, the relationship between m6A methylation modification and esophageal cancer still needs to be further explored. Based on m6A-related genes, several previous studies have constructed riskscore that can stably predict the prognosis of patients with multiple cancer types, including ESCC (17, 18). But different from other studies, our study analyzed m6a-related genes from the perspective of single-cell mapping for the first time, and constructed a riskscore that stably predicts the prognosis of ESCC patients.

Therefore, we investigated the microscopic roles of the major pathways of m6A methylation and differential genes in esophageal carcinogenesis and invasive metastasis in ESCC patient tumors. Single cell sequencing and cell communication analysis were used to clarify the spatially specific major biological functions of the distribution of m6A methylation-related genes in tumor cells. Transcriptomics and single-cell sequencing revealed that subpopulations of tumor cells with differential expression of m6A methylation-related gene profiles were heterogeneously distributed within the lesion. m6A methylation differential expression correlated significantly with the immune infiltration status of EACC patients. This suggests that the aberrant distribution of m6A methylation may determine poor prognosis and immune tolerance in ESCC patients. To enrich the clinical scalability of the model, we assessed the specific relationship between m6A methylation-related genes and immune infiltration and constructed subgroups to assess their impact on drug sensitivity. These findings provide new insights into the spatial biology and immunological understanding of m6A methylation in ESCC, and make some breakthroughs for individualised treatment and new target development in ESCC.

## Materials and methods

### Data collection

All patient data in this study were obtained from online public databases, including the cancer genome atlas (tcga) and GEO DataSets. All patients included in the study had complete public gene expression data and clinical annotation. A bulk RNA-seq of 77 patients with esophageal squamous carcinoma from the tcga-ESCC cohort was included, while gene expression data containing samples of esophageal cancer tissue and normal esophageal tissue were downloaded from the gene expression omnibus (GEO) data (http://www.ncbi.nlm.nih.gov/geo/). GSE53625, a core data set of expression profiles, was used for validation. It includes bulk RNA-seq from 174 patients with esophageal squamous carcinoma and single cell scRNA-seq data from GSE188900 from 4 patients with esophageal squamous carcinoma. The clinical data collected included age, gender, tumor stage and prognostic data. To ensure the availability and reliability of the data, strict inclusion and exclusion criteria were established for this study. Inclusion criteria: (1) primary squamous cell carcinoma of the esophagus confirmed by pathological sections; (2) patients with a prognostic follow-up of at least 30 days; (3) complete genomic expression level data included; and (4) clear reporting of pathological conditions and follow-up. Exclusion criteria: (1) other pathological types or secondary esophageal tumors; (2) concurrent primary tumors from other sites.

### Collection of genes associated with epigenetic modifications of m6A and single-cell data processing

In this study, 21 m6A regulators were collected from the MSigDB database, including 8 writers (METTL3, METTL14, RBM15, RBM15B, WTAP, KIAA1429, CBLL1, ZC3H13), 2 erasers (ALKBH5, FTO) and 11 readers (YTHDC1, YTHDC2, YTHDF1, YTHDF2, YTHDF3, IGF2BP1, HNRNPA2B1, HNRNPC, FMR1, LRPPRC, ELAVL1). The original expression profile dataset (GSE188900) used for the analysis was filtered from the public database GEO database platform. The data set was 5 EACC tumor specimens, which were extracted from RNA and analyzed by expression profiling microarray, using the Illumina NextSeq detection platform. The raw data set was pre-processed with the Seurat R package to ensure quality control (QC) results. The number of genes detected in each cell (nFeature_RNA) and the total number of molecules detected within the cell (nCount_RNA) were calculated, and the number of genes detected per cell was proportional to the number of genes expressed (reads) obtained by sequencing. Cells were clustered based on the filtered principal components and visualized using uniform manifold approximation and projection (UMAP) dimensionality reduction techniques for cell classification. Tumour cell marker genes with adj. p value < 0.05 were screened. Cell marker genes were retrieved using the PanglaoDB database, and the genes corresponding to each class cluster were intersected to determine the class cluster in which the cells were located. Cell population grouping was performed by single cell sequencing data acquisition post-processing and downscaling analysis. This includes T cells, B cells, Mast cells, fibroblasts, myeloid and endothelial cells and the remaining cell types.

### Analysis of protein interactions and cellular communication of m6A epigenetic modifications in ESCC at the single cell level

After pre-processing and downscaling analysis of single-cell scRNA-seq data from four patients with esophageal squamous carcinoma from GSE188900, PROGENy scores were calculated to demonstrate the scoring of tumor-associated pathways in different cell populations.PROGENy (Pathway RespOnsive GENes for activity inference) is an R package published in 2018. It is generally accepted that gene expression correlates with pathway signaling activity, and in previous pathway enrichment analyzes this has largely been used as a basis for assuming that the more genes highly expressed in a pathway, the more likely it is that the pathway is activated, however, the effect of post-translational modifications has been ignored. Based on this, PROGENy can use publicly available data from perturbation experiments to infer common core genes in the pathway response of human samples. This can be used to infer pathway activity from bulk RNAseq science. This fits in with our research aim to understand the m6A epigenetic gene pathway alterations in single cell samples from ESCC. Also, Cell Chat scores were calculated for different species of immune cells sequenced from single cells, demonstrating cellular communication for each cell population. Cellular communication analysis can help us understand cell-cell interactions and resolve intercellular communication networks. It will help us to unravel the interactions of various cell types during development, explore the immune microenvironment of tumors and uncover potential therapeutic targets for diseases. CellChat has built a cellular interaction database of 2021 ligand-receptor pairs that can be used to quantify intercellular communication networks from single cell transcriptome data, resolve the major input and output signals for each cell type, and suggest how each cell type and multiple signaling pathways work together. Based on the fact that the macrophage migration inhibitory factor (MIF) signaling pathway is the secretory signaling pathway with the highest probability of communication in ESCC cells, the cellular communication network of the ‘MIF’ pathway is demonstrated.

### Gene enrichment and functional analysis of the m6A epigenetic pathway

The m6A signature (m6A epigenetic) was scored for gene set enrichment using the AddModuleScore function of the Seruat package, and was divided into two groups: m6A signature-high and m6A signature-low. It can be seen that most of the immune cells have a mixture of m6A signature-high and m6A signature-low groups. Further analysis was carried out in the Bulk transcriptome. The R package “LIMMA” (version 3.48.3) was used to compare the two groups. LMFIT and bayes functions were used to ensure accuracy. The R packages clusterProfiler, org.Hs.eg.db, DOSE, enrichment plot, and colourspace were used for gene enrichment and pathway functional analysis of DEGs and core genes. The clusterProfiler R package was used to perform gene ontologyGO) functional enrichment analysis and Kyoto encyclopedia of genes and genomes (KEGG) pathway enrichment analysis on the differential gene sets. The enrichment analysis is based on the principle of hypergeometric distribution, where GO is a comprehensive database describing gene function and can be divided into 3 parts: biological process (BP), cellular component (CC) and molecular function (MF). KEGG is a comprehensive database that integrates genomic, chemical and systemic functional information. We want to analyze the key macroscopic associations of m6A epigenetically related genes and signaling pathways during development at the cellular component, molecular function and biological process levels. Enrichment pathways are all significant at p value < 0.05. In addition, we used the “Limma” package to find out the differential genes between the two groups based on the “Ebays” function, and performed a univariate Cox regression analysis on the differentially expressed genes (DEGs) based on the tinyarray package, with a p value < 0.01, and 29 genes were selected.

### m6A epigenetic survival-related differential gene acquisition and prognostic model construction

To further visualise as well as quantify the potential impact of m6A epigenetic inheritance on the development of ESCC patients. We performed univariate Cox regression analysis on the 29 DEGs obtained in the previous step, and selected prognostic genes with p value < 0.05 as the screening criterion. The cohort GSE53625 of 174 patients with squamous esophageal cancer was also used for independent external validation. We divided ESCC patients into high-risk and low-risk groups by the median riskscore. Also, Kaplan-Meier (K-M) survival curves were plotted for the high- and low-risk groups. Combining common clinical parameters and riskscore to draw a nomogram for further clinical visualization and auxiliary application of the model.

### Exploring the relationship between the role of m6A epigenetics in immune infiltration and the impact of treatment sensitivity in ESCC

Clarifying the immune infiltration of m6A epigenetics in the tumor microenvironment and the lymphocyte association is an important prerequisite for understanding its impact on treatment resistance in ESCC patients. To ensure predictive accuracy, we used ssGSEA and xCell algorithm (19) to assess the level of immune infiltration. Based on the expression matrix, xCell evaluates the composition and abundance of immune cells in mixed cells by combining the advantages of deconvolution methods and gene enrichment. Based on the expression matrix and the immune cell marker gene set, ssGSEA can calculate enrichment scores for individual samples and gene set pairs to determine the level of immune infiltration. ssGSEA is able to quantify the abundance of immune cells and stromal cells from transcriptomic data to assess the level of immunity one at a time. In addition, immune scores and tumor purity were calculated for each sample by the ESTIMATE algorithm.

In view of the above studies showing the close association of m6A epigenetics for immune infiltration in ESCC, it is considered that m6A may interfere with immune infiltration in ESCC by interfering with tumor immunity against chemotherapy. Further, we wanted to analyze whether the therapeutic susceptibility of ESCC patients to multiple chemotherapeutic agents is definitively associated with the m6A epigenetic pathway, in order to facilitate the clinical dissemination and application of the model in the future. Here, we used the Cancer Genome Project (CGP) database to predict the therapeutic susceptibility of chemotherapeutic agents using the R package “prophet”. After initial analysis of the data, we used the Genomics of drug sensitivity in Cancer (GDSC) database to calculate drug sensitivity scores using the R package ‘oncopredict’ (20) and visualised the predicted images using multiple box plots.

### Tissue acquisition and quantitative real-time PCR

Ten pairs of ESCC tissues and adjacent normal tissues were collected from Shandong Provincial Hospital of Shandong University and stored at -80°C for a long time. RNAiso Plus (Takara Bio, Japan) was used to extract total RNA from tissues, PrimeScript RT Master Mix (Takara Bio, Japan) was used for reverse transcription of cDNA, and TB green (Takara Bio, Japan) was used for RT-qPCR reaction. and normalized with GAPDH. All primer sequences are detailed in **Supplementary Table 1**.

### Statistical analysis

Independent t-tests and Mann-Whitney U tests were used to compare two groups of variables with normal and non-normal distributions, respectively, and to determine statistical significance. One-way ANOVA (analysis of variance) and Krush-Wallis tests were used to compare differences between multiple sets of statistics. RT-qPCR data were compared using Student’s t-test. All statistical analyzes were performed using R software for statistical analysis. Statistical significance was defined as a p value < 0.05.

## Results

### Preliminary visualization and distribution analysis of single-cell sequencing data from ESCC

**Figure 1:**
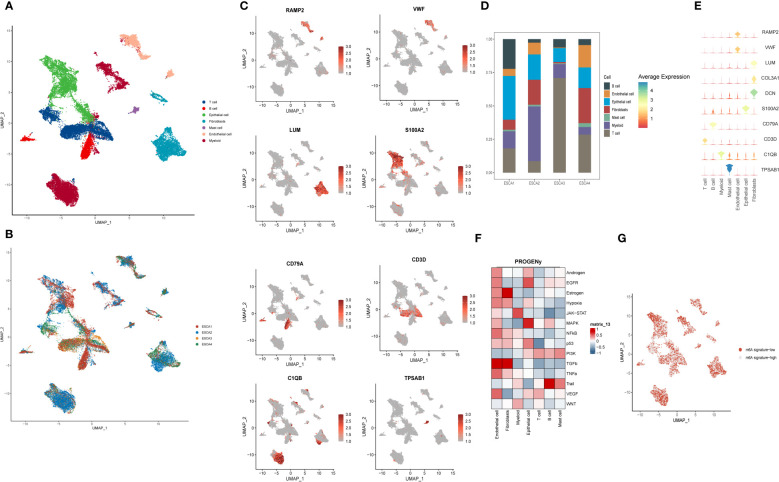
Spatial depiction of single-cell sequencing data and annotation of immune cell types and gene distribution in ESCC patients. **(A)** U-MAP plot showing the sample source of the single-cell data, with each colour representing one sample, including a total of 4 patients; **(B)** Representative 7 major immune cell types and distribution annotations in ESCC cells, including T cells, B cells, epithelial cells, fibroblasts, mast cells, endothelial cells and myeloid cells; **(C)** Single-cell sequencing obtained from 7 cell types in the major m6A gene cluster expression distribution maps, including RAMP2, VWF, S100A2, LUM, CD79A and CD3D. outlines the spatial distribution of expression of their major immune cell types. **(D)** Scale bar graphs further analyze the distribution of immune cell types in four different ESCC patients; **(E)** Bubble plots further visualize the major m6A epitopes obtained in **(B)** 7 major immune cell types and distribution annotated expression relationships; **(F)** Heat map showing the expression of tumor-associated pathway scores for different immune cell populations obtained by the PROGENy method; **(G)** Single-cell sequencing visualization of m6A signature for gene set enrichment scoring, divided into m6A signature-high and m6A signature-low groups.

First, single-cell analysis was performed on scRNA-seq data from four esophageal squamous carcinoma samples from GSE188900, downlinked into seven cell clusters. Preliminary in-expression normalization was performed and we classified the cells into coherent transcriptional clusters using a graph-based clustering approach. UMAP method downlink analysis was performed to group the cells into clusters. The main categories of immune cell clusters identified and annotated included:Tcell, B cell, Epithelial, Fibrobalst, Mast Cell, Endotheli and Myeloid (**Figure 1A**), and cells were annotated according to the source of the sample. Furthermore, single-cell sequencing data obtained from four ESCC patients were annotated for spatial distribution and classified primarily based primarily on differences in the taxonomic content of their immune cells and associated signaling pathway alterations. These were ESCA1, ESCA2, ESCA3 and ESCA4, respectively (**Figure 1B**). The images yielded that ESCA1 and ESCA2 patients were labelled with more single cell sequencing data and expressed higher content. In contrast, for ESCA3 and ESCA4 patients, relatively low levels of single cell expression were annotated. Cells were annotated by clusters and clusters were classified into seven main categories. By combining the cellular distribution of the multi-locus single cell transcriptome profiles, we found that the annotated expression of endothelial cells was high, mainly in ESCA1, ESCA2 and ESCA3 patients, located throughout the top right side of the image. myeloid cells were mostly distributed in the ESCA3 patient population, located in the lower part of the image. Fibroblasts are mainly annotated in ESCA2 patients and are located in the lower left of the image. B cells and Mast cells received relatively few cellular annotations.

We wanted to gain further insight into the genetic correlation between the expression of m6 epigenetic-related genes in single-cell data and their distribution in different sequencing groups. We first analyzed the expression distribution of 8 immune cell-specific marker genes in 7 types of immune cells (**Figure 1C**). RAMP2 and VWF were highly expressed in endothelial cells; Epithelial mainly expressed S100A2, fibroblasts expressed LUM, CD79A and CD3D were expressed in B cells and T cells respectively, and C1QB was mainly located in Myeloid cells. tPSAB1 gene was less expressed. In **Figure 1D**, we applied a scale bar chart to further analyze the distribution of immune cell types in four different ESCC patients. In ESCA1 patients, Epithelial cells were found to be more expressed in about 30% of the patients, in addition to mainly T cells and B cells. For all four patients, Mast cells were relatively less expressed, which is similar to our perception of the distribution of immune space. For ESCA2, Myeoloid cells were the main component, with Epithelial cells and fibroblasts dominating the distribution. In contrast, ESCA3 is dominated by T cells. For ESCA4, the distribution of the content of the various types of immune cells was more uniform. In **Figure 1E**, we further visualised the distribution of the main m6A epigenetic immune cell types obtained in **Figure 1B** through bubble plots. Further, we used PROGENy to show the tumor-related pathway scores of the different cell populations (**Figure 1F**). Endothelial cells were highly positively correlated with the expression of various tumor cell pathways, the most active of which was the TGFb pathway, considering that they may be mainly related to ESCC invasion and metastasis. Fibroblasts mainly expressed Estrogen and TGFb pathways. The tumor pathways of Epithelial cells are mainly enriched in EGFR and MAPK, and the significant enrichment of these two ‘star pathways’ may suggest a significant role of Epithelial cells in the development of ESCC. For T cells, all tumor pathways were not significantly enriched. In contrast, B cells showed a significant positive correlation with Trail pathway expression. In addition, the m6A signature was scored for gene set enrichment by function into two groups: m6A signature-high and m6A signature-low. It was concluded in **Figure 1G** that the majority of immune cells were present in a mixture of m6A signature-high and m6A signature-low groups, suggesting the need for further quantitative studies for analysis.

Further, we studied single cell sequencing data and interactions between different immune cells from ESCC patients through cellular communication. CellChat score is an open source R package (http://github.com/sqjin/CellChat) that allows the use of scRNA-seq data to infer, visualize and analyze intercellular communication. As shown in **Figure S1A**, T cells and B cells interact primarily with Epithelial cells. Epithelial cells send signals that are primarily associated with T cells. Fibroblasts are more closely associated with T cells and Epithelial cells. Mast cells send signals focused on T cells. Endothelial cells and Myeloid cells interacted mainly with T cells. In addition, we calculated the interaction of four signaling pathway networks, MIF, AFF, FN1 and CD99, as shown in **Figure S1B**. In the MIF signaling pathway network, T cells and B cells were the main signal sourcing as well as signal receivers, suggesting their main immune regulatory role in the MIF network. For the APP signaling network, Endothelial cells are the main source of signaling, targeting a wide range of immune cells. Similarly, in FN1, the major signaling pathway for fibrosis, fibroblasts play a more dominant role, sending signals targeting a variety of immune cell types. In contrast, for the CD99 signaling pathway network, the interaction of signaling pathways across cell types is more complex and there is no key cell type that is more deterministic.

### Gene enrichment and pathway functional analysis of m6A epigenetic inheritance

**Figure 2:**
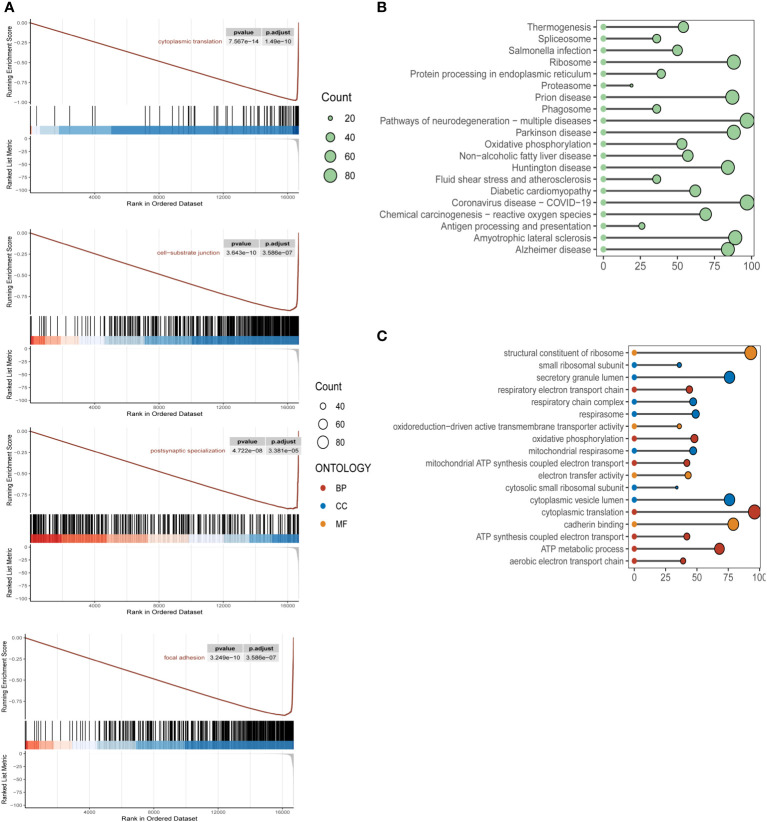
Biological functional enrichment analysis of m6A epigenetic in ESCC single cell data **(A)**. KEGG enrichment pathway analysis of m6A epigenetic-related genes in ESCC patients, mainly enriched in the functional features of cytoplasmic translation, cell-substrate junction, postsynaptic specialization, focal adhesion **(B)**. GSEA pathway analysis of m6A epigenetically related genes in ESCC patients; **(C)** GO pathway analysis of m6A epigenetically related genes in ESCC patients.

To clarify the specific implications and potential biological functions of the macroscopic role of m6A epigenetics in the development of ESCC, we used GSEA, KEGG and GO analysis to enrich for markers and pathway functions. In the GSEA analysis (**Figure 2A**), signaling pathways were mainly enriched in cytoplasmic translation, cell-substrate junction, postsynaptic specialization, and focal adhesion. In the GO analysis (**Figure 2B**), the main m6A epigenetic pathways were expressed in the structural constituent of Oxidative phosphorylation, Proteasome, ribosome, Protein processing in endoplasmic reticulum, etc. In the KEGG pathways of m6A epigenetics in ESCC patients were mainly enriched in structural constituent of ribosome, cytoplasmic translation, secretory granule lumen, cadherin binding, etc (**Figure 2C**).

### Acquisition of survival-associated differential genes for m6A epigenetic inheritance and construction and validation of prognostic models

**Figure 3:**
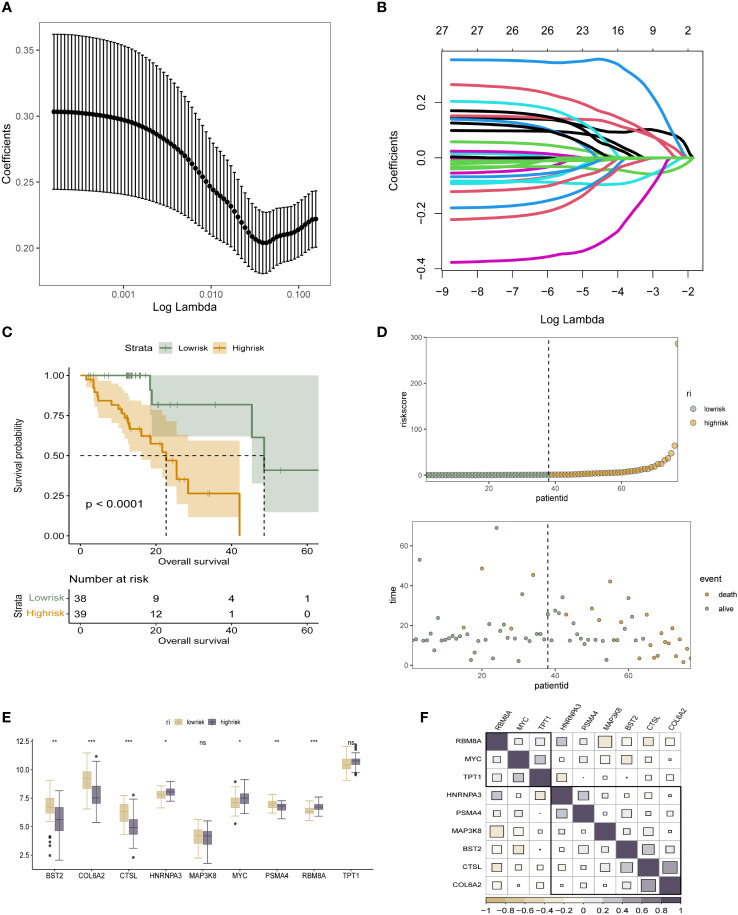
Acquisition of survival-associated differential genes for m6A epigenetic inheritance and prognostic model construction **(A, B)**. LASSO regression process for screening survival-related prognostic genes from 29 m6A epigenetically related differential genes; **(C)**. K-M analysis reveals the prognostic stratification ability of the constructed m6A epigenetically related riskscore for patients in the high-risk and low-risk groups, respectively; **(D)**. Dotted line and scatter plots reveal the different riskscore of survival time and survival status of ESCC patients, from left to right representing the high and low variation of riskscore, respectively, and scatter colours representing survival and death status; **(E)**. Box line plot revealing the expression distribution of 10 risk genes in the low- and high-risk groups of the 6A epigenetic patient population; **(F)**. Expression correlation plot of the 10 risk genes constituting the riskscore. * means <0.05, ** means <0.01, *** means <0.001, ns means >0.05.

Subsequently, to further clarify the direct association of m6A epigenetically related genes with survival in ESCC patients directly, We obtained m6A-signature differential genes and screened out the prognostic genes among them. Using the TCGA-ESCC cohort, nine out of 29 genes were selected based on Lasso regression to build a prognostic model. The specific process of LASSO regression and the coefficient selection process are shown in **Figures 3A, B**. The risk genes screened included BST2, COL6A2, CTSL, HNRNPA3, MAP3K8, MYC, PSMA4, RBM8A, TPT1. and riskscore were constructed by linearly multiplying the screened risk genes by linear multiplication to distinguish the low-risk group from the high-risk group by the median of the riskscore to build to clarify the stratification of the constructed riskscore on the prognosis of ESCC patients, we analyzed the survival status of different m6A epigenetic subgroups using K-M curves. A significant difference in survival prognosis between patients with high as well as low expression of m6A epigenetic markers can be seen in **Figure 3C**. In addition, bubble and scatter plots were used to further visualise the survival prognosis of the different riskscore patients, as shown in **Figure 3D**. Further, we also depicted the distribution of expression of nine risk genes in the m6A epigenetic low- and high-risk groups of the patient population (**Figure 3E**). The results showed that, except for RBM8A, TPT1 and HNRNPA3, all m6A epigenetically inherited genes were highly expressed in the low-risk group, suggesting that they were the main protective genes. In **Figure 3F**, we analyzed the expression correlations of the nine risk genes constituting the riskscore, and the results showed that the expression among RBM8A, MYC, and TPT1 genes possessed a high positive correlation. The expression of HNRNPA3, PSMA4, MAP3K8, BST2, CTSL and COL6A2 were more closely associated with each other.

**Figure 4:**
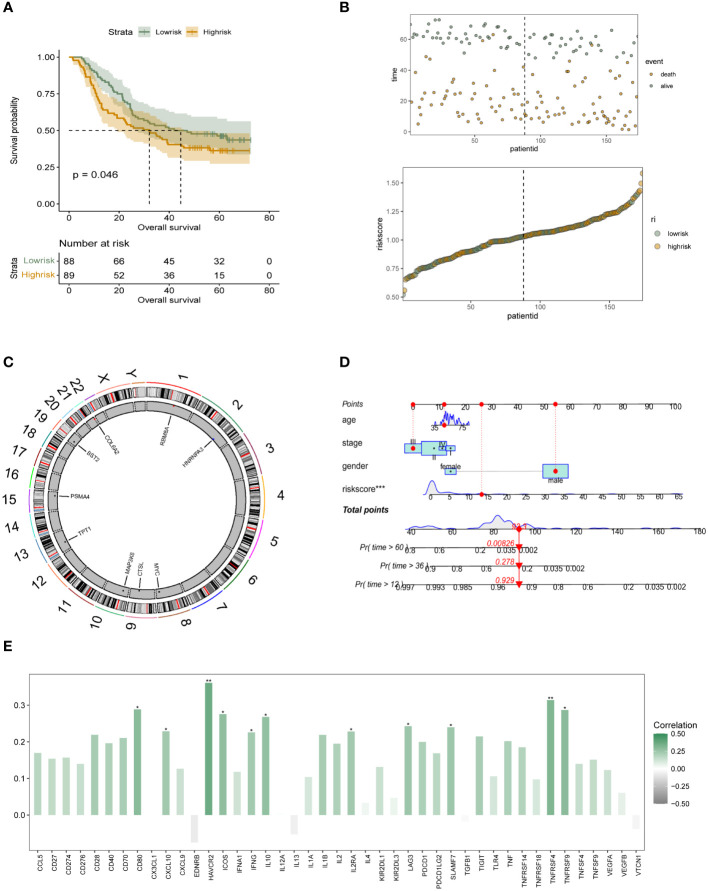
Independent validation and initial immune-related characterization of the survival risk model for m6A epigenetics **(A)** K-M analysis in GEO’s independent validation database reveals the prognostic stratification ability of the constructed m6A epigenetic-associated riskscore for patients in the high-risk and low-risk groups, respectively; **(B)** Dotted line and scatter plots reveal the survival time and survival status of ESCC patients with different riskscore, from left to right, representing the riskscore of high and low variation, and the scatter colours represent survival and death status; **(C)** Chromosomal expression location map of m6A genes constituting the risk model; **(D)** Nomogram based on m6A riskscore reveals the prognostic stratification score of ESCC patients; **(E)** Bar graphs initially show the potential association between m6A epigenetic and tumor immune infiltration-related proteins. * means <0.05, ** means <0.01, *** means <0.001.

In the 174 patients with esophageal squamous carcinoma in cohort GSE53625, we further performed independent external validation of the constructed m6A epigenetic riskscore. In **Figure 4A**, we performed survival analysis and survival curve validation in the independent validation group. The results show that our constructed riskscore is able to significantly differentiate the survival status of ESCC patients, with patients with high m6A riskscore having a significantly lower prognostic survival status than those in the low-risk group. We also applied scatter plots and dotted line plots to depict the distribution of m6A riskscore among patients in the low- and high-risk groups, as shown in **Figure 4B**. This suggests that the riskscore we constructed has significant prognostic stratification predictive power in both patient groups with ESCC. In more depth, we wanted to understand the distribution of the m6A epigenetic gene. In **Figure 4C** it is shown that RB8MA and HNRNPA3 are located on chromosomes 1 and 2, respectively, and that MYC, CTSL and MAP3K8 are expressed on chromosomes 8, 9 and 10. tPT1 and PSMA4 are distributed on chromosomes 14 and 15. bst2 and COL6A2 are distributed on chromosomes 19 and 22, respectively. The results showed that the genomic localization of m6A-related genes was scattered and did not show obvious clustering. In order to further facilitate the clinical use of riskscore and to combine the decision-making features of integrated clinical multifaceted analysis and judgment. We constructed a Nomogram for model visualization based on m6A epigenetic and other clinicopathological factors (**Figure 4D**). The Nomogram, consisting of age, Stage, Gender, and riskscore, significantly predicted the prognosis of ESCC patients in a stratified manner. In addition, we also preliminarily explored the correlation of m6A epigenetic score with the expression of immune-related signalling pathways and signalling molecules. In addition, we performed correlation analysis on riskscore and immune modulator analysis. The results showed that CD80, HAVCR2, ICOS, IL10, TNFRSF4 and multiple immune-related pathway proteins were significantly positively correlated with the expression of riskscore (**Figure 4E**). This tentatively revealed a potential association between m6A epigenetics and tumor immune infiltration.

### Assessment of the role of m6A epigenetics in immune infiltration in ESCC

**Figure 5:**
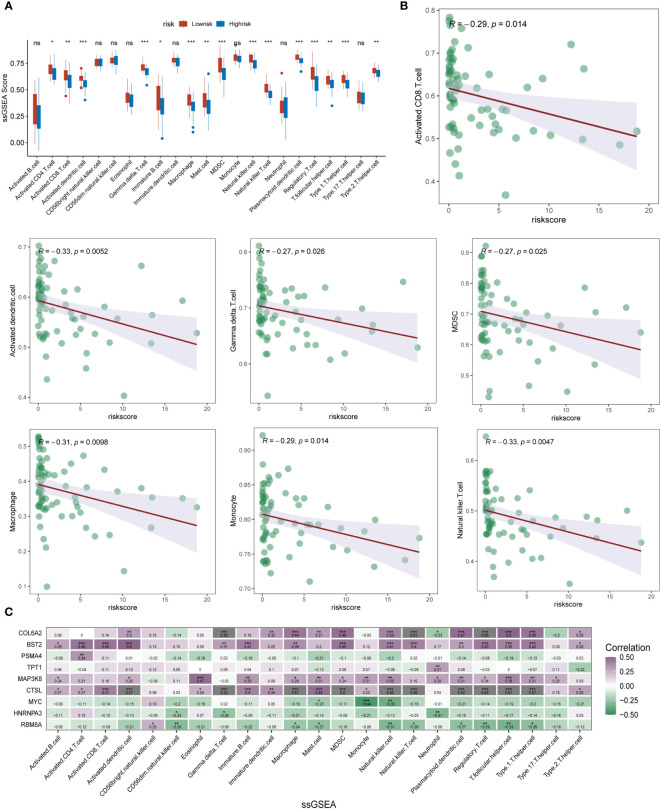
ssGSEA analysis of immune infiltration and cell expression annotation in ESCC patients. **(A)** Box plot format analyzing the differential expression of multiple immune cells and immune-related proteins in the m6A high-risk and low-risk groups. The expression of several types of immune cells, including activated CD8 T cells, MDSC, gama delta T cells, activated dendritic cells, NK T cells, macrophages, and Monocyte, differed significantly between subgroups, and the m6A epistasis The expression levels of these immune cell types were significantly higher in the low-risk group than in the high-risk group; **(B)** Linear correlation plots assessed the expression correlations between multiple immune cell types and the riskscore; **(C)** Correlational heat map analysis further clarified the correlation between multiple immune cells and immune infiltration levels and the expression of each specific gene in the m6A risk model (shades of colour indicate the level of correlation, purple indicates positive correlation, blue indicates negative correlation). * means <0.05, ** means <0.01, *** means <0.001, ns means >0.05.

To further determine the direct association of lysosomal pathway-related risk genes with the specificity of immune infiltration and immune cell secretion in ESCC, we calculated immune infiltration scores using two methods, ssGSEA and xCell, visualized by box plot, heat map and scatter plot, respectively. The results of immune infiltration analyzed by the ssGSEA method were first analyzed. In **Figure 5A**, we analyzed the differential expression of multiple immune cells and immune-related proteins in the m6A high-risk and low-risk groups, and presented them in the form of box plots. We noted that the expression of activated CD8T cells, MDSC, gama delta T cells, activated dendritic cells, NK T cells, macrophages and Monocyte were significantly different between the different subgroups, and the expression levels of these immune cells were significantly higher in patients in the m6A epigenetic low-risk group than in those in the high-risk group. This suggests that m6A may have a potential impact on tumor development, mainly by affecting the expression of immune cells and their immune function. Further, we analyzed the expression correlation between several of the previously proposed cell types and the riskscore. The specific characteristics of the associations were analyzed by means of linear correlation plots, as shown in **Figure 5B**. The results showed that multiple immune cells were negatively correlated in expression with increased riskscore, p value < 0.05. In **Figure 5C**, we further clarified the expression correlation of multiple immune cells and immune infiltration levels with specific each gene in the m6A risk model using correlation heat map analysis. Here we found that COL6A2, BST2, TPT1, and MAP3K8 were predominantly positively correlated with high expression of immune cells. In contrast, for the other genes that make up the m6A epistatic risk model, including CTSL, PSMA4, MYC, HNRNPA3, and RB8MA, there was a negative correlation with the majority of differentially expressed immune cells. In addition, we further clarified and multi-methodologically confirmed this differential expression relationship using the xCell method (**Figure S2A-S2C**). The results likewise showed that the m6A epigenetic-based riskscore was significantly correlated with multiple immune cell types, with ESCC patients with higher riskscore having lower expression levels of their major immune cells, with this difference being reflected mainly in NKT, CD4+ naive T cells, M1 macrophages, aDC, and macrophages.

### Assessing the role of m6A epigenetics in ESCC treatment sensitivity

**Figure 6:**
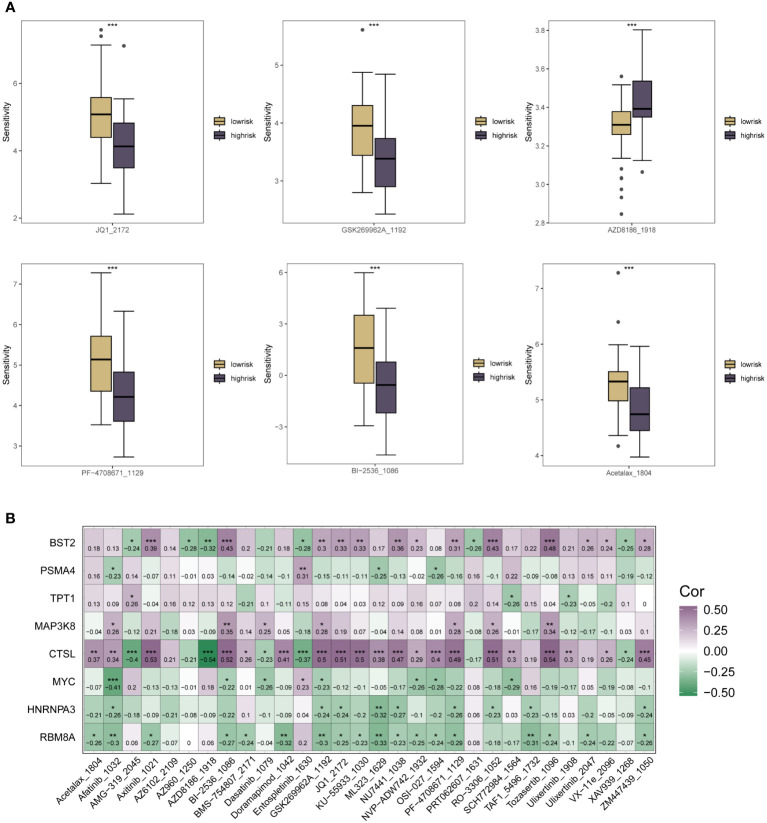
Correlation analysis of the riskscore expression of m6A in ESCC patients with the sensitivity scores of drugs in the GDSC database. **(A)** Box plots of specific expression associations between treatment sensitivity and riskscore groupings for multiple oncology therapies. Chemotherapeutic agents include AZD8186_1918, SK269962A_1192, Acetalax_1804, Bl-2536_1086, JQ1_2172, PF-4708671_1129; **(B)** Correlation heat map analysis further clarifies the therapeutic sensitivity of multiple chemotherapeutic agents with specific each gene in the m6A risk model for expression correlation. * means <0.05, ** means <0.01, *** means <0.001.

As a result of the immune infiltration analysis described above and the multi-method functional evaluation of different immune cell types, we have been able to understand the specific patterns of association between the m6A methylation risk model and tumor immunity and prognosis in ESCC patients. Several clinical trials have shown that current ESCC immunotherapy is less effective, and to explore whether m6A could be helpful in this pathway in ESCC, specific associations between the therapeutic sensitivity of various oncology therapies and the riskscore subgroup were assessed. Sensitivity scores for drugs in the GDSC database were calculated based on the R package “oncoppredict”. As shown in **Figure 6A**, for chemotherapeutic agents such as AZD8186_1918, patients in the low-risk group of the m6A riskscore had significantly lower treatment sensitivity than those in the high-risk group. In contrast, for chemotherapeutic agents such as GSK269962A_1192, Acetalax_1804, Bl-2536_1086, JQ1_2172 and PF-4708671_1129, patients in the m6A low-risk group had higher therapeutic sensitivity, which may be related to the close m6A epigenetic in tumor immune infiltration and regulation of multiple immune cells association. In **Figure 6B**, we used correlation heat map analysis to further clarify the correlation between the therapeutic sensitivity of multiple chemotherapeutic agents and the expression of each specific gene in the m6A risk model. Here we found that CTSL, BST2, PSMA4, TPT1 and MAP3K8 were predominantly positively correlated with the therapeutic sensitivity of multiple chemotherapeutic agents. In contrast, for the other genes that make up the m6A epistatic risk model, including COL6A2, MYC, HNRNPA3, and RB8MA, there was a resistance correlation with the majority of chemotherapeutic drug treatment effects.

### Expression verification of risk genes

**Figure 7:**
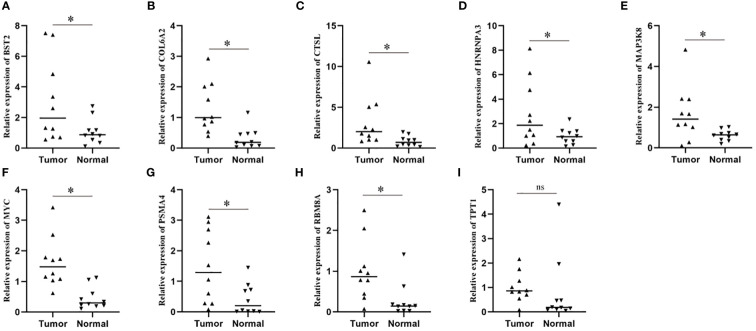
Expression levels of model genes in a real-world cohort. **(A–I)** qPCR assay was used to detect the transcription levels of model genes in tissue samples. * means <0.05.

In order to further explore the expression of risk genes in ESCC tissues, we verified the expression of risk genes in ESCC tissues and adjacent normal tissues using RT-qPCR. The results showed that, except for TPT1, there was no difference in expression between cancer and paracancerous tissues, and the expression of the other 8 risk genes in ESCC tissues was significantly higher than that in paracancerous normal tissues (**Figures 7A–I**).

## Discussions

In recent years, although the incidence and mortality rates of esophageal cancer have been significantly reduced, the overall survival rate of esophageal cancer patients after surgery is still low and the prognosis is poor. The study of the molecular biology of esophageal cancer development can provide new targeted therapeutic strategies for the precise treatment of esophageal cancer and improve the prognosis and overall survival time of patients, and is therefore a hot research topic in the field of esophageal cancer (21, 22). m6A methylated modification, as one of the most common RNA modifications, can regulate gene expression at the post-transcriptional level, and in recent years its related enzymes or proteins have also been investigated for their role in the development of esophageal cancer. In recent years, the function of its related enzymes or proteins in the development of esophageal carcinoma has also been widely investigated. Studies (23) have shown that m6A methylation-modified binding protein is specifically highly expressed in a variety of tumors and that its upregulated expression promotes tumor proliferation, migration and invasion. However, the mechanism of its role in esophageal cancer remains unclear. In this study, we investigated the specific mechanism of m6A epigenetic development in esophageal cancer and clarified the immune cell distribution of m6A-related genes in ESCC tumor microenvironment by single cell sequencing analysis. Meanwhile, m6A differential genes were selected to construct a risk model to achieve a sensitive stratification of ESCC patients’ prognosis. The association of m6A epigenetics with immune infiltration and abnormal expression of immune cells was also investigated by various methods. Finally, the specific impact of differential m6A expression in esophageal cancer in ESCC on tumor chemotherapy resistance was analyzed.

Related studies have found that m6A, as the most abundant epigenomic modification in eukaryotic cells, plays an important role in tumorigenesis and progression (24). Studies have shown that among hundreds of known RNA modifications, m6 A methylation modification is the most common internal modification in messenger RNA (mRNA), affecting RNA shearing, translation, stability and epigenetic effects of certain non-coding RNAs. In mammalian cells, there are on average one to two m6 A methylation sites per 1,000 nucleotides (25). m6 A methylation regulators have been shown to be aberrantly expressed in a variety of tumors and play an important role in the regulation of malignant biological behaviours such as cell proliferation, invasion and metastasis (26), and are involved in the development and progression of various cancers such as leukaemia, glioblastoma, lung cancer and liver cancer (27). In this paper, we explored and described the composition and mode of action of m6 A modification regulators, their biological functions in the disease progression of esophageal cancer, as well as the prognostic value and potential clinical applications of m6 A methylation modification in esophageal cancer, providing some entry angles for in-depth exploration of the mechanism of esophageal cancer development and the search for esophageal cancer biomarkers and therapeutic targets. In our study, we used the ESCC single-cell mapping to perform enrichment scoring according to m6a-related genes, and divided them into m6a signature-high and m6a signature-low. And the differential genes among different m6a features were analyzed. We performed further pathway and biological function enrichment analysis of the screened DEGs. The results revealed that m6A was mainly associated with cytoplasmic translation, cell-substrate junction, Thermogenesis, structural constituent of ribosome. This suggests that m6A may influence the development of ESCC and cell mutation through chromosome structural composition pathway and cellular structural reorganization, providing a new way to investigate the specific mechanism of m6A in ESCC. In addition, the study also found a specific association between differential m6A expression and tumor immune infiltration and abnormal expression of immune cells, suggesting that m6A interferes with tumor immune pathways to influence treatment resistance in ESCC.

This study also has some limitations. Firstly, as a retrospective analysis, there are limitations in terms of data acquisition. Secondly, the study design was biased in terms of variable selection. Information on important molecular factors such as HER2/neu overexpression was not included in the data analysis (28, 29). These factors have been shown to be associated with the prognosis of patients with ESCC. Also, the database does not contain complete treatment records, such as information on the choice of chemotherapy regimen or targeted therapy (30). Furthermore, some factors of laboratory tests, such as tumor markers, are important for survival of cancer patients (31) and the authors do not have sufficient knowledge of these factors. These advances will be incorporated into these important factors to improve the prognostic value of future m6A-related esophageal cancer studies.

In our study, a blueprint for the single-cell distribution of ESCC based on m6A methylation was explored, and on this basis risk models were constructed for immune infiltration analysis and tumor efficacy stratification in ESCC. m6A methylation plays a close role in tumor developmental invasion, but there is a lack of relevant studies reported in ESCC. Therefore, this may play an important potential guide to reveal the role played by m6A for immune escape and treatment resistance in esophageal cancer.

## Data availability statement

The datasets presented in this study can be found in online repositories. The names of the repository/repositories and accession number(s) can be found in the article/**Supplementary Material**.

## Ethics statement

Studies involving human subjects were reviewed and approved by the Ethics Committee of Shandong Provincial Hospital of Shandong University. Patients/participants provided written informed consent to participate in this study.

## Author contributions

Manuscript writing: YN; Review and correct: FZ, ZY; data collection: GY. All authors contributed to the article and approved the submitted version. 
