# Supplementary material for: Single-cell mapping of N6-methyladenosine in esophageal squamous cell carcinoma and exploration of the risk model for immune infiltration
Source: Front Endocrinol (Lausanne). 2023 Mar 21;14:1155009. doi: 10.3389/fendo.2023.1155009 (PMC10070687; doi:10.3389/fendo.2023.1155009)
Supplement: Supplementary file 1 [file DataSheet_1.zip › ETHICS CERTIFICATE OF APPROVAL.pdf]

山东大学附属省立医院医学伦理委员会
涉及人的生物医学研究项目伦理审批件

省医伦批第（LCYJ:NO. 2019-090）

项目名称	同步放化疗联合 PD-1 免疫抑制剂治疗局部晚期食管鳞癌单臂探索性研究				
项目负责人	杨哲	职　称	主任医师	科室	
项目来源	自筹经费				
审查文件	■伦理审查申请书　■研究方案　■知情同意书（模板）　■快审批件<br>■涉及药品的临床研究项目备案表　□使用医疗技术的临床研究项目备案表<br>□其他资料________				

伦理审查意见

Δ 同意	同意
Δ 修改后同意	
Δ 不同意（项目终止或暂停）	

审批意见

1.经本伦理委员会审查，同意进行该项目临床研究。意见和建议：■无　□有

2.伦理委员会对该研究实施过程的年度/定期跟踪审查：■是　□否

审查期限为研究批准之日起：□三个月　□六个月　■十二个月

3.伦理委员会有权根据实际进展情况改变年度/定期跟踪审查期限。

4.自批准之日起半年内项目未启动，该批件自动失效。

山东大学附属省立医院医学伦理委员会

（盖章）

2019年 5月31日

HUAWEI Mate 20 Pro
LEICA TRIPLE CAMERA | AI
