# Supplementary material for: Single-cell mapping of N6-methyladenosine in esophageal squamous cell carcinoma and exploration of the risk model for immune infiltration
Source: Front Endocrinol (Lausanne). 2023 Mar 21;14:1155009. doi: 10.3389/fendo.2023.1155009 (PMC10070687; doi:10.3389/fendo.2023.1155009)
Supplement: Supplementary file 2 [file DataSheet_2.docx]

**Single-cell mapping of N6-methyladenosine in esophageal squamous cell carcinoma and exploration of the risk model for immune infiltration**

**Supplementary Information**

**Figure S1. Single-cell sequencing data on immune cell communication and signaling pathway network interactions in ESCC patients.**

A. Cellular communication and interaction maps of seven major immune cell types in representative ESCC cells, including T cells, B cells, epithelial cells, fibroblasts, mast cells, endothelial cells and myeloid cells; B. Signal sources and targeting interactions of seven major immune cell types in four key tumour signalling pathway networks, including MIF, AFF The four major tumour signalling pathways include MIF, AFF, FN1 and CD99.


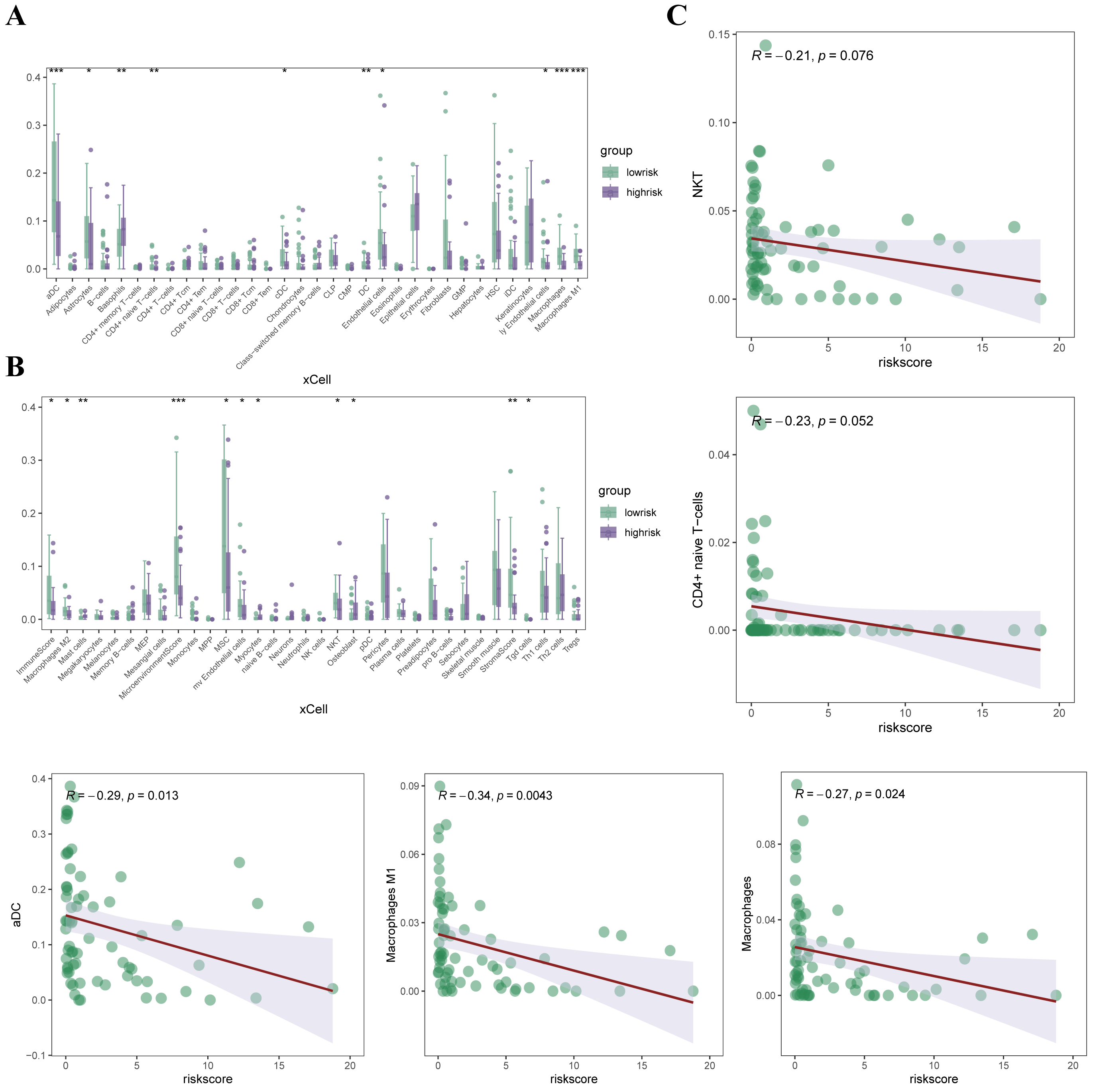


**Figure S2. xCELL method for immune infiltration analysis and cell expression annotation in ESCC patients.**

A. Box plot format analyzing the differential expression of multiple immune cells and immune-related proteins in the m6A high-risk and low-risk groups. m6A epigenetic low-risk patients had significantly higher expression levels of multiple immune cells than those in the high-risk group; B. Box plot analyzing the differential expression of multiple immune-related pathways in the m6A high-risk and low-risk groups; C. Linear correlation plot evaluating the multiple immune cell types and expression correlations between riskscore, with COL6A2, BST2, TPT1, and MAP3K8 showing a predominantly positive correlation with high expression of immune cells. In contrast, for the other genes comprising the m6A epistatic risk model, including CTSL, PSMA4, MYC, HNRNPA3, and RB8MA, there was a negative correlation with the majority of differentially expressed immune cells.
